# Influence of Information Sources on Chinese Parents Regarding COVID-19 Vaccination for Children: An Online Survey

**DOI:** 10.3390/ijerph19127037

**Published:** 2022-06-08

**Authors:** Kai Li, Fen Zhou

**Affiliations:** School of Journalism and Communication, Guangxi University, Nanning 530004, China; 2021391064@st.gxu.edu.cn

**Keywords:** COVID-19 vaccination, parents’ intention to vaccinate children, social media, information sources exposure

## Abstract

(1) Aims: This study explored the mechanism by which exposure to different information sources on social media influences Chinese parents’ intention to vaccinate their children against COVID-19. (2) Methods: We developed a research framework based on the Stimulus–Organism–Response (SOR) theory to illustrate how exposure to information sources on social media increases vaccine confidence and, as a result, parents’ intentions regarding pediatric vaccination. The partial least square structural equation modeling (PLS-SEM) method was used to test the data collected through an online survey (687 valid samples). (3) Results: The government approval of vaccines fuels vaccination confidence and acts as a mediator between (a) mass media, government new media, and key opinion leaders, and (b) perceived effectiveness and side effects (safety) of vaccines. (4) Conclusions: The mass media, government new media, and key opinion leaders are crucial sources for encouraging parents to vaccinate their children since they boost the vaccination trust. The focus of COVID-19 vaccination promotion should be to strengthen parents’ trust in the government, combined with publicizing the effectiveness and side effects (safety) of vaccines.

## 1. Introduction

Many health professionals believe that the long-term answer to the coronavirus (COVID-19) is to achieve herd immunity through widespread vaccination [1]. Since 2021, certain global pharmaceutical companies have produced and commercialized a variety of children’s COVID-19 vaccinations [2,3]. According to the National Health Commission of the People’s Republic of China (NHC), Chinese children’s infection rate of COVID-19 has increased since 2021, and the incidence of the disease in children is close to that of adults. In addition, severe or fatal cases occur frequently among children, so they also need vaccine protection. COVID-19 vaccines approved for emergency use by children (aged 3–17 years) in China are inactivated vaccines, including the Sinopharm COVID-19 vaccine (Beijing), Sinopharm COVID-19 vaccine (Wuhan), and Sinovac COVID-19 vaccine [4]. Health experts claim that children who are not vaccinated are more likely to compromise the overall pandemic prevention efforts [5]. The invention of the COVID-19 vaccines—one of the most rapidly manufactured vaccines for infectious disease in human history—is a remarkable achievement [6]. However, individuals continue to be concerned about the vaccines’ safety, efficiency, and long-term durability of protection. This public concern leads to vaccination hesitation, refusal [7,8,9], and impediments to herd immunity [10]. Therefore, convincing parents to vaccinate children remains a major challenge [11].

Previous research has found a correlation between social media and vaccination confidence [12,13]. Furthermore, a few existing studies assert that the quality of vaccination-related discourse on social media varies, and that negative, misleading, and anti-vaccine information appears to raise parents’ concerns [14,15,16]. In contrast, many other suggest that social media help promote immunizations. According to the research of Daley and Glanz, strategic communication through social media encourages vaccinations and enhances parents’ positive perception [17,18], so that social media can be leveraged as a valuable tool to boost COVID-19 immunization [19]. The remaining crucial question regards the factors and interventions that significantly increase parents’ vaccination confidence [19,20].

China hopes to end the COVID-19 pandemic by creating herd immunity, with youngsters playing a pivotal role [21]. In June 2021, the government approved COVID-19 immunization for children aged 3 to 17 [4], but persuading Chinese parents to vaccinate their children remains a significant challenge. Additionally, the public perceives COVID-19 information mainly through social media in China [22], but very few efforts have examined how social media affects parental acceptance of COVID-19 vaccination for children. Notably, Wang et al. approached this issue from families with a medical background [23], and Zhang gauged the effect merely from the social media content regarding parental acceptability [13]. The scarcity of studies in this field constrains our understanding of COVID-19 vaccination promotion. Due to the principles of “least effort” and “cost-benefit” [24], in which people intend to seek information sources at low costs [25], social media must be subject to a broader range of interrogation. As such, this study asked the following questions:

Question 1: Which social media information sources impact parents’ intentions to vaccinate their children against COVID-19?

Question 2: What is the mechanism of the effect of exposure to information sources on parents’ intentions to vaccinate their children against COVID-19?

This research has a threefold contribution. First, it expands our understanding of public health communication by looking into the effects of social media information sources on parents’ intentions of pediatric vaccination. Second, it helps deepen our understanding of the psychological mechanism by which exposure to information sources on social media influences Chinese parents’ intentions to vaccinate their children. Third, the findings from this study practically aid the promotion campaign of children’s COVID-19 immunizations.

### 1.1. Theoretical Foundation

The SOR model, proposed by Mehrabian and Russell, reflects the effect of the environmental stimulus on individual’s awareness and response [26]. In the SOR model, S refers to the stimulus from the external environment; O indicates that the individual processes the stimulus information perceived from external sources according to the existing cognition; and R refers to the individual’s response after forming a subjective cognition by receiving and processing the stimulus information [26]. The SOR model has become one of the most commonly used models to study human information behavior, and it allows us to better understand the relationship between external stimuli and individual responses.

#### 1.1.1. Information Sources as Stimulus

Social media comprise the main channel for the public to obtain information related to COVID-19 vaccines [22]. Matthew et al. noted that social media can significantly influence parents’ attitudes toward vaccination for children [17]. Glanz noted that exposure to different information sources can influence parents’ intentions to vaccinate their children [18]. People also consider information from different sources before making decisions [27,28]. A literature review by Clarke et al. indicated that health information sources include technology-based sources, traditional mass media, and human sources, namely close social ties [29]. In China, the information sources on social media include government new media, mass media, key opinion leaders, professional organizations, and personal networks [30]. “Government new media” refers to the new media accounts of the government and its subordinate agencies, e.g., the WeChat official accounts and microblog accounts of the publicity department of the Party committee, government departments and Centers for Disease Control (CDC). “Mass media” refers to the social accounts of traditional media such as newspapers, radio, television, and magazines. “Key opinion leaders” are experts in the health care field, e.g., Nanshan Zhong (academician of Chinese Academy of Sciences), Wenhong Zhang (doctor of Huashan Hospital affiliated to Fudan University), and Lanjuan Li (academician of Chinese Academy of Engineering). “Professional organizations” refers to social media accounts opened by professional organizations or companies in the health field, such as the World Health Organization (WHO), and media companies that promulgate science and technology (Dr. Dingxiang). “Personal networks” refers to an individual’s social relationships, such as family, friends, and colleagues. Therefore, the frequency of parents’ exposure to these types of information sources was used as a stimulus in this study.

#### 1.1.2. Vaccine Confidence as Organism

Health experts have stated that individual attitudes toward vaccination are fundamentally related to vaccine confidence [31,32], and that building trust and increasing confidence are keys to promoting vaccination [33]. According to the Strategic Advisory Group of Experts on Immunization of WHO, vaccine confidence can be divided into three aspects: trust in government approval of vaccine, trust in vaccine delivery systems, and vaccine trust [34,35]. The development, production, storage and transportation, and use of COVID-19 vaccines in China are all served by companies or institutions with governmental backgrounds. Therefore, this study integrated trust in the government approval of vaccines and trust in vaccine delivery systems into trust in the government approval of vaccines. Thus, vaccine confidence includes trust in government approval of vaccines and trust in vaccines. Wang [35] and Paul [36] argued that trust in vaccines includes trust in vaccine effectiveness and trust in vaccine side effects (safety). Bell [37] and Szilagyi et al. [38] noted that effectiveness and side effects (safety) are the most important concerns of parents or guardians for the COVID-19 vaccines. Accordingly, this study further classified vaccine confidence into three aspects: trust in government approval of vaccines, perceived effectiveness of vaccines, and side effects (safety) of vaccines.

#### 1.1.3. Vaccination Intentions as Response

The health action process approach suggests that “intention” is an important predictor of “behavior” [39]. Ashkenazi and Glanz have noted that exposure to information sources influences parents’ intentions to vaccinate their children [18,40]. Therefore, this study used vaccination intentions as the dependent variable.

### 1.2. Research Hypotheses and Model

Previous studies showed that an individual’s exposure to information sources significantly affects his or her vaccine confidence [32]. Studies in India, Bangladesh, and Nigeria found that parents who are consistently exposed to positive vaccine-related information through mass media have greater confidence in vaccines [41]. Studies in Georgia [42], Indonesia [43], Taiwan [44], and Canada [45] showed that frequent exposure to information such as errors, questions, and negative event reports weakens parents’ confidence in vaccines.

In China, mass media and government new media are important channels for Chinese government to promote COVID-19 vaccination. Famous KOLs, such as Nanshan Zhong and Wenhong Zhang, and China’s leading research institutes, are supporters of the vaccination. According to NHC, by 26 August 2021, over 800 million people were voluntarily vaccinated [46]. Overall, support for vaccination is high within China. In summary, it is speculated that the frequency of exposure to information sources above is positively correlated with parents’ confidence in vaccines. Therefore, it was hypothesized that:

**Hypothesis** **1** **(H1a).** *Frequency of exposure to “government new media” is positively related to trust in government approval of vaccine.*

**Hypothesis** **1** **(H1b).** *Frequency of exposure to “mass media” is positively related to trust in government approval of vaccine.*

**Hypothesis** **1** **(H1c).** *Frequency of exposure to “key opinion leaders” is positively related to trust in government approval of vaccine.*

**Hypothesis** **1** **(H1d).** *Frequency of exposure to “professional organizations” is positively related to trust in government approval of vaccine.*

**Hypothesis** **1** **(H1e).** *Frequency of exposure to “personal networks” is positively related to trust in government approval of vaccine.*

**Hypothesis** **2** **(H2a).** *Frequency of exposure to “government new media” is positively related to perceived effectiveness of vaccine.*

**Hypothesis** **2** **(H2b).** *Frequency of exposure to “mass media” is positively related to perceived effectiveness of vaccine.*

**Hypothesis** **2** **(H2c).** *Frequency of exposure to “key opinion leaders” is positively related to perceived effectiveness of vaccine.*

**Hypothesis** **2** **(H2d).** *Frequency of exposure to “professional organizations” is positively related to perceived effectiveness of vaccine.*

**Hypothesis** **2** **(H2e).** *Frequency of exposure to “personal networks” is positively related to perceived effectiveness of vaccine.*

**Hypothesis** **3** **(H3a).** *Frequency of exposure to “government new media” is positively related to side effects (safety) of vaccines.*

**Hypothesis** **3** **(H3b).** *Frequency of exposure to “mass media” is positively related to side effects (safety) of vaccines.*

**Hypothesis** **3** **(H3c).** *Frequency of exposure to “key opinion leaders” is positively related to side effects (safety) of vaccines.*

**Hypothesis** **3** **(H3d).** *Frequency of exposure to “professional organizations” is positively related to side effects (safety) of vaccines.*

**Hypothesis** **3** **(H3e).** *Frequency of exposure to “personal networks” is positively related to side effects (safety) of vaccines.*

Previous studies showed that, the more individuals trust the government during the COVID-19 pandemic, the more they trust the government approval of vaccines [47], and the effectiveness and side effects (safety) of vaccines [35,36]. Conversely, the less individuals trust the government, the less they trust the government approval of vaccines, and the effectiveness and side effects (safety) of vaccines [48,49]. Based on this, it was hypothesized that:

**Hypothesis** **4** **(H4).** *Trust in government approval of vaccines is positively related to the perceived effectiveness of vaccines.*

**Hypothesis** **5** **(H5).** *Trust in government approval of vaccines is positively related to side effects (safety) of vaccines.*

Bell et al. noted that the approval of COVID-19 vaccines by the government, or their recommendation by official health care providers, is one of the main factors to improve parents’ intentions of vaccinating children [37]. Latkin indicated that the more individuals trust the government, the more likely they are to accept the COVID-19 vaccination [49]. Karlsson’s findings showed that approximately three-quarters of the respondents were willing to accept government approval of vaccines [50]. Therefore, it was hypothesized that:

**Hypothesis** **6** **(H6).** *Trust in government approval of vaccines is positively related to vaccination intentions.*

The study by Meltem et al. noted that the main reason parents have their children vaccinated against COVID-19 is that the vaccine prevents COVID-19 (effectiveness) and is safe [51]. Emphasizing the safety and effectiveness may be more effective in increasing public acceptance when communicating new solutions (such as COVID-19 vaccines) to health risks [52]. Therefore, it was hypothesized that:

**Hypothesis** **7** **(H7).** *Perceived effectiveness of vaccines is positively related to vaccination intentions.*

**Hypothesis** **8** **(H8).** *Side effects (safety) of vaccines are positively related to vaccination intentions*.

**Figure 1:**
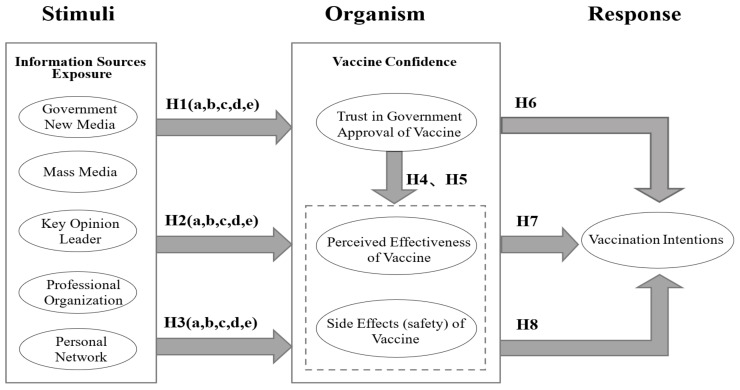
Research model based on the SOR model.

In conclusion, the model of this study is shown in Figure 1.

## 2. Materials and Methods

### 2.1. Participants and Data Collection

The study was conducted in the form of a web-based questionnaire by www.wjx.cn (accessed on 1 September 2021), a platform similar to Google Forms. Chinese parents (with at least one child aged 3–17) were invited to fill out the questionnaire. The sample was chosen through the “charged sample” service offered by www.wjx.cn (accessed on 1 September 2021) with a sample library of 0.3 billion people. Data were collected from 23 August to 31 August 2021. Finally, a total of 808 samples were collected, and after deleting incomplete questionnaires, 687 valid questionnaires were obtained, with a valid recovery rate of 85%. The distribution of the participants is presented in Table 1.

### 2.2. Measures

First, to identify information sources that may influence parents’ willingness to vaccinate their children, we conducted interviews with 6 parents. Interview questions included: Whether you plan to have your child vaccinated against COVID-19? Whether information on social media influences your decision to vaccinate your child against COVID-19? From which information sources (accounts) do you get related information? Having obtained a first draft of the list of information sources, we collated the information sources’ names to create a formal list according to the definition of the China Internet Network Information Center (CNNIC) report [30]. Then, the list was used in the “frequency of information sources exposure” section of the questionnaire.

Items measuring vaccine confidence, including trust in government approval of vaccine, perceived effectiveness of vaccines, and side effects (safety) of vaccines, and items measuring parents’ intentions to vaccinate their children, were all taken from the questionnaire designed by Paul et al. [36], which has been used in previous studies.

The independent variable, frequency of exposure to information sources, was measured by a four-level scale (from 1 = “never” to 4 = “always”), and trust in government approval of vaccines, perceived effectiveness of vaccines, side effects (safety) of vaccines, and vaccination intentions were measured using a five-level Likert scale (from 1 = “strongly disagree” to 5 = “strongly agree”). The above data and demographic characteristic items (gender, education, age, etc.; see Table 1 for details) were combined to form the pre-study questionnaire. We invited a public health expert and a vaccine specialist to review the draft version of the questionnaire, and then modified its wording according to their advice.

For the pilot-test, we conducted convenience sampling in which 50 parents (with at least one child aged 3–17) from Nanning, Guangxi, completed the questionnaire. The reliability and validity of the variables were calculated based on the pilot-test data. In the reliability test, Cronbach’s α of each variable was higher than 0.7. In the validity test, factor loadings of all measurement dimensions on the variables and cross-loadings of all measurement dimensions on other variables were examined through factor analysis, and the results showed good validity. Therefore, the final questionnaire was determined for this study; see Appendix A for details.

## 3. Data Analysis and Results

In this study, the independent variable, frequency of exposure to information sources, is a formative indicator, and the variables of trust in government approval of vaccines, perceived effectiveness of vaccines, side effects (safety) of vaccines, and vaccination intentions are reflective indicators. Moreover, this study is an exploratory study with a complex model structure. Therefore, we used partial least square structural equation modeling (PLS-SEM) to validate the model, and processed the data in SmartPLS 3.3.3 (SmartPLS GmbH, Oststeinbek, Germany).

PLS-SEM analysis takes place in two steps. First, the measurement model is tested to ensure the quality of constructs used in the model through reliability and validity testing (details are given in Section 3.1. Measurement Model Testing). Second, structural model assessment is carried out to examine the relationship between the constructs using the path coefficient (β) and coefficient of determination (R^2^). For significance testing, the complete bootstrapping procedure was run with 5000 samples. We followed the guidelines of Hair et al. (2016) for evaluation and reporting results [52].

### 3.1. Measurement Model Testing

#### 3.1.1. Reflective Construct

Assessment of the reliability, convergent validity, and discriminant validity of the reflective constructs was undertaken as follows: first, the reliability was assessed using Cronbach’s α and composite reliability (CR) [53]. The measurement results are shown in Table 2. Both Cronbach’s α and CR are higher than 0.8, indicating good reliability. Second, convergent validity was assessed. As seen in Table 2, all item loadings are higher than 0.70, and all average variance extracted (AVE) values are higher than 0.50, suggesting convergent validity [53]. Finally, discriminant validity was assessed and the results are shown in Table 3. Values of the diagonal of the matrix are the square roots of AVE, and values of the non-diagonal are the correlation coefficients between the cross-sections. The square roots of AVE are higher than the correlation coefficients, which is a good indication of discriminant validity [53]. Table 2 and Table 3 confirm the reliability and validity of the reflective constructs involved in the study. In the final model, trust in government approval of vaccines was measured with seven items, perceived effectiveness of vaccines with four items, side effects (safety) of vaccines with four items, and vaccination intentions with two items.

#### 3.1.2. Formative Constructs

The quality of formative constructs was assessed by examining collinearity, the significance of both outer weights, and item loadings on the given constructs [53]. The results of the formative indicator testing are shown in Table 4. The variance inflation factor (VIF) should be between 0.2 and 5. The VIF for all the formative items was between 1 and 1.48, showing no issue of collinearity. When evaluating the significance of formative items, the significance (*p*-value) of outer weights is first checked, then the item loadings, and finally the *p*-value of item loadings, if required. In case the outer weights are not significant, item loadings are checked; a check for the *p*-value of the item loadings is needed if item loadings are under 0.5. Items with high loadings are retained, and others are deleted from further analysis. Of nine formative items for exposure to information sources, only two items (PO3 and PO4) from professional organizations could not fulfill the quality criteria for formative constructs. The outer weights of PO3 and PO4 were less than 0.2, i.e., loadings of PO3 and PO4 were less than 0.5. Hence, PO3 and PO4 were removed. After the measurement model assessment, government new media and professional organizations were each measured with two items.

### 3.2. Structural Model Testing

**Figure 2:**
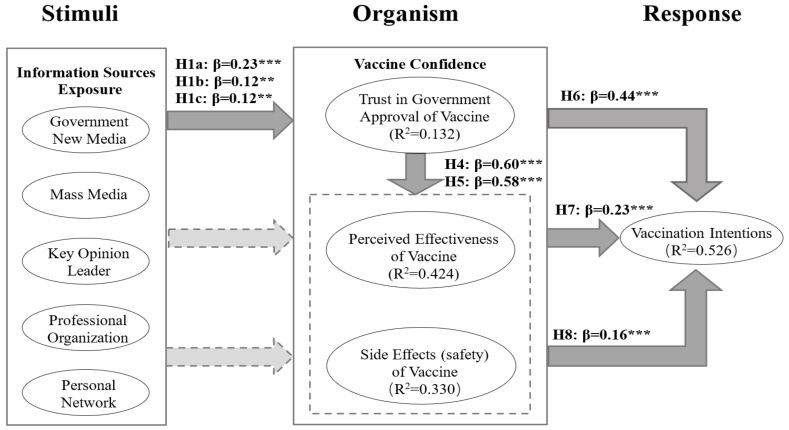
Results of the research model. (a) **: *p* < 0.01, ***: *p* < 0.001. (b) Straight arrows indicate significant direct effects; dotted arrows indicate non-significant direct effects.

This study first examined the path coefficients (β) and the coefficients of the determination (R^2^) using the PLS algorithm, and then tested the significance of the model by bootstrapping (subsamples = 5000) [53]. We examined the effect of exposure to information sources on vaccine confidence, the effect of vaccine confidence on vaccination intentions, and the effect of trust in government approval of vaccines on perceived effectiveness of vaccines and side effects (safety) of vaccines. Figure 2 shows the path coefficients and coefficients of determination for trust in government approval of vaccines, perceived effectiveness of vaccines, side effects (safety) of vaccines, and vaccination intentions. For complete statistics, consult Table 5.

The results of Figure 2 and Table 5 are as follows. When examining the relationship of the exposure to information sources and vaccination confidence, H1a (β = 0.23 *p* < 0.001), H1b (β = 0.12 *p* < 0.01), H1c (β = 0.12; *p* < 0.01) could be substantiated. Other hypotheses, i.e., H1 (d, e), H2 (a, b, c, d, e), and H3 (a, b, c, d, e), could not be substantiated. Exposure to information sources accounted for 13.2% of the variance in trust in government approval of vaccines. For the relationship of trust in government approval of vaccines to perceived effectiveness of vaccines and side effects (safety) of vaccines, H4 (β = 0.60 *p* < 0.001) and H5 (β = 0.58 *p* < 0.001) could be substantiated. Trust in government approval of vaccines accounted for 42.4% of the variance in perceived effectiveness of vaccines. Trust in government approval of vaccines accounted for 33.0% of the variance in side effects (safety) of vaccines. The relationship of vaccine confidence to vaccination intentions provided evidence to support H6 (β = 0.44 *p* < 0.001), H7 (β = 0.23 *p* < 0.001), and H8 (β = 0.16 *p* < 0.001). Trust in government approval of vaccines, perceived effectiveness of vaccines, and side effects (safety) of vaccines, accounted for 52.6% of the variance in vaccination intentions.

## 4. Discussion

### 4.1. Key Findings

Based on SOR theory, this study constructed an analytical framework for different types of information sources on social media that vary in terms of the influences of Chinese parents’ intentions to vaccinate their children. The key findings are as follows.

Vaccine confidence is primarily shaped by exposure to government new media, mass media, and key opinion leaders, and, consequently, affects parents’ plans regarding pediatric vaccination. There are no discernible consequences from exposure to professional organizations or personal networks. The government new media refers to state official information channels and social media accounts. In other words, the public considers the two categories of information sources associated with the CCP committee’s media operation to be authoritative and reliable. Meanwhile, key opinion leaders perform well in social media. This finding is on contrast to Allington et al., who argue that traditional media exposure has a beneficial effect on the public’s intentions regarding vaccination, while social media had no meaningful impact [54]. Our research reinforces the finding that social media has an essential impact on public perceptions regarding COVID-19 immunization, which differs for various information sources. Evaluating social media as an overall subject without sub-categorization may further cause a cognitive bias. Therefore, methodologically, the constructed framework to differentiate distinct parts of social media is essential.

Regarding the impact of information sources on the trust in government approval of vaccines, three categories of information sources—the mass media, government new media, and key opinion leaders—indicate a significant positive correlation. The other two components of vaccination confidence, namely vaccine effectiveness and side effects (safety), show no correlation with the information sources. This contradicts the findings of Hohmeier et al. [55] and Huang [47], who purport that personal network exposure (community physicians, community pharmacists, and community opinion leaders) signifies a positive impact on vaccine effectiveness and safety. Furthermore, this study finds that trust in vaccine approval by the government relates to parents’ perceived efficacy and side effects (safety), and is very likely to increase the overall acceptability rate of pediatric vaccination.

The findings from this study provide an opposite view to that of a few existing literature studies due to the difference in country. The Chinese public seems to place a high value on government-related information providers [48]. Such sources generally show a direct, considerable impact on parents’ trust in vaccination. However, the existing studies in other countries indicate a different scenario; for example, American people obtain vaccine information from trusted interpersonal ties such as familiar professionals and community opinion leaders [36].

Based on these two findings, we speculated that trust in government approval of vaccines may act as a mediating variable between exposure to information sources and perceived effectiveness and side effects (safety) of vaccines. To this end, we also tested the mediating role of trust in government approval of vaccines (as shown in Table 6 [52]). The results confirmed that trust in government approval of vaccines acts as a mediating variable between exposure to information sources and perceived effectiveness and side effects (safety) of vaccines. That is, trust in government approval of vaccines mediates the effects of exposure to information sources on the perceived effectiveness of vaccines and side effects (safety) of vaccines. Previous research has indicated that concerns about the vaccine’s side effects (safety) and effectiveness are the primary reasons for the public to delay or even refuse inoculation [48]. It is difficult to remove public reservations regarding the COVID-19 vaccines’ side effects (safety) and effectiveness only through the media, given the vaccines’ development and implementation in such a short period of time. Due to the delicate and fragile nature of children, it is more difficult to convince parents of the safety and efficacy of vaccinations. The Chinese people have a high level of trust in the central government [48], and, for parents, products that have been approved by the government provide assurances of safety and efficacy.

Trust in government approval of vaccines, perceived effectiveness of vaccines, and side effects (safety) of vaccines can all directly and significantly influence parents’ intentions to vaccinate their children against COVID-19. Among these factors, trust in government approval of vaccines has the most significant effect on COVID-19 vaccination intentions, and perceived effectiveness of vaccines and side effects (safety) of vaccines have secondary effects. Our findings differ from the conclusions of Chu and Liu [52,56], which emphasize that safety and effectiveness of vaccines may be more effective in increasing public acceptance of vaccines. We believe that the findings are also the result of different levels of trust in government in different countries or regions [48].

### 4.2. Implications of Study

The study’s findings have a variety of implications. First, as the survey discovered that key opinion leaders, mass media, and government new media positively affect parents’ trust and intentions about COVID-19 immunization, we may target information sources for vaccine communication promotion campaigns, for instance, by maximizing the coverage and reach of these three types of sources. Second, parents’ conviction in the government’s approval of vaccines is a critical factor in their faith in the vaccine’s efficacy and safety, and their intention to vaccinate their children. As a result, building parents’ trust in government approval of vaccines must be the government’s top priority in promoting pediatric vaccination. Third, this study examined the effect of information sources on Chinese parents’ intentions to vaccinate their children against COVID-19, and serves as a case study in China for pediatric vaccine promotion that can be used as a model for other countries.

### 4.3. Limitations and Future Research

It is crucial to acknowledge the study’s limitations. First, all data for the variables were self-reported by participants, and may thus involve subjective bias, even though self-reporting is a common strategy used in this field to measure variables. Second, data were gathered via an online questionnaire that may entail methodological biases (e.g., sample size and response bias) and limit the generalizability of the study outcome. Third, the design of this study was cross-sectional. It was not possible to monitor the temporal evolution and trajectory of respondents’ vaccination intentions, nor could causal linkages be established. Fourth, the study mainly explored the influence of exposure to different information sources, and ignored the influence of the content of the information source. Despite these limitations, we believe that this study makes a significant contribution to the field by elucidating the mechanism by which various information sources on social media affect parents’ intentions to vaccinate their children against COVID-19. Additional research can go further by following these trajectories: first, conducting a separate study using probability sampling; second, increasing the prediction potential of the study model by incorporating more constructs; and third, examining the temporal effect with longitudinal research. Finally, we strongly recommend that future studies be conducted on the influence of content related to COVID-19 vaccines on parents.

## 5. Conclusions

This research sheds light on the manner in which exposure to information sources on social media affects Chinese parents’ decisions to vaccinate their children against COVID-19. The study found that mass media, government new media, and key opinion leaders are important information sources for reinforcing Chinese parents’ confidence in vaccines and, as a result, their intentions regarding pediatric vaccination. These research results may help in designing targeted strategies to promote pediatric vaccination. The focus of COVID-19 vaccination promotion should be to improve parents’ trust in the government, combined with publicizing the effectiveness and side effects (safety) of the vaccine. Healthcare practitioners can use these data to discover vaccination motivators in their practice and emphasize the critical nature of immunization.

## Figures and Tables

**Table 1:** Demographic profile of participants (*n* = 687).

Characteristic		Count	%
Gender	Male	314	45.7
	Female	373	54.3
Age	18–30	151	22.0
	31–40	454	66.1
	41 or above	82	11.9
Education	Junior high school or below	9	1.3
	Senior high school or equivalent	40	5.8
	Undergraduate and College Degree	521	75.8
	Postgraduate	117	17.0
Type of work	Frontline workers	308	44.8
	Management staff	300	43.7
	Freelancers	67	9.8
	Unemployed	1	0.1
	Home makers	10	1.5
	Student	1	0.1
Relationship’s status	Married	662	96.3
	Currently single or divorced	19	2.8
	Having a stable boyfriend or girlfriend	6	0.9
Having at least one family member with a history of COVID-19	Yes	18	2.6
	No	669	97.4
Friend/Neighbor/Colleague diagnosed with COVID-19	Yes	30	4.4
	No	657	95.6
Number of the children	1	498	72.5
	2	178	25.9
	3	11	1.6

**Table 2:** Results for the measurement models.

Constructs	Items	Mean	SD	Loadings	α	CR	AVE
Mass Media (MM)	MM	3.38	0.72	1.00	1.00	1.00	1.00
Key Opinion Leader (KOL)	KOL	2.97	0.84	1.00	1.00	1.00	1.00
Personal Network (PN)	PN	2.98	0.83	1.00	1.00	1.00	1.00
Trust in Government Approval of Vaccine (TRUST)	TRUST 1	4.36	0.62	0.80	0.89	0.91	0.60
	TRUST 2	4.25	0.71	0.73			
	TRUST 3	4.20	0.72	0.76			
	TRUST 4	4.49	0.64	0.78			
	TRUST 5	4.40	0.68	0.75			
	TRUST 6	4.30	0.68	0.80			
	TRUST 7	4.36	0.69	0.80			
Perceived Effectiveness of Vaccine (EFF)	EFF 1	4.37	0.67	0.80	0.80	0.87	0.62
	EFF 2	4.24	0.75	0.76			
	EFF 3	4.27	0.79	0.78			
	EFF 4	4.24	0.76	0.81			
Side Effects(safety) of Vaccine (SAV)	SAV 1	4.17	0.82	0.80	0.90	0.93	0.77
	SAV 2	3.52	1.08	0.91			
	SAV 3	3.48	1.11	0.91			
	SAV 4	3.62	1.07	0.90			
Vaccination Intentions (VI)	VI 1	4.39	0.74	0.94	0.85	0.93	0.87
	VI 2	4.38	0.71	0.93			

SD = Standard Deviation; α = Cronbach’s α.

**Table 3:** The divergent validity matrix of the research model using the Fornell–Larker method.

Constructs	EFF	SAV	TRUST	VI
EFF	0.79			
SAV	0.54	0.88		
TRUST	0.64	0.57	0.77	
VI	0.60	0.54	0.68	0.93

(a) The diagonal elements are the square roots of the AVE values; (b) the non-diagonal elements are the squared correlations among factors.

**Table 4:** Results of collinearity, weights, and item loadings.

Constructs		Mean	SD	VIF	Weights	*p*	Loadings	*p*
Government New Media (GNM)	GNM1	3.16	0.86	1.45	0.47	0.00	0.84	0.00
	GNM2	3.13	0.86	1.45	0.66	0.00	0.92	0.00
Professional Organization (PO)	PO1	2.37	0.91	1.40	0.41	0.15	0.70	0.00
	PO2	2.66	0.93	1.46	0.80	0.00	0.94	0.00
	*PO3*	*2.58*	*0.98*	*1.32*	*0.05*	*0.85*	*0.47*	*0.02*
	*PO4*	*2.02*	*0.90*	*1.48*	*−0.14*	*0.57*	*0.42*	*0.02*

(a) SD = Standard Deviation; (b) insignificant factors are shown in italic.

**Table 5:** Results of hypothesis testing.

Hypotheses	Relationship	β	T	*p*	Results
H1a	GNN → TRUST	0.23	4.88	0.00	Supported
H1b	MM → TRUST	0.12	2.82	0.01	Supported
H1c	KOL → TRUST	0.12	2.50	0.01	Supported
H1d	PO → TRUST	−0.06	1.03	0.31	Not Supported
H1e	PN → TRUST	0.06	1.56	0.12	Not Supported
H2a	GNN → EFF	0.03	0.72	0.47	Not Supported
H2b	MM → EFF	0.02	0.54	0.59	Not Supported
H2c	KOL → EFF	0.07	1.81	0.07	Not Supported
H2d	PO → EFF	0.02	0.52	0.60	Not Supported
H2e	PN → EFF	0.06	1.82	0.07	Not Supported
H3a	GNN → SAV	0.04	0.97	0.33	Not Supported
H3b	MM → SAV	−0.05	1.37	0.17	Not Supported
H3c	KOL → SAV	−0.04	0.92	0.36	Not Supported
H3d	PO → SAV	0.02	0.55	0.59	Not Supported
H3e	PN → SAV	−0.03	0.90	0.37	Not Supported
H4	TRUST → EFF	0.60	16.82	0.00	Supported
H5	TRUST → SAV	0.58	18.18	0.00	Supported
H6	TRUST → VI	0.44	4.83	0.00	Supported
H7	EFF → VI	0.23	8.93	0.00	Supported
H8	SAV → VI	0.16	4.16	0.00	Supported

**Table 6:** Results of mediation analysis.

Independent Variable	Intermediary Variable	Dependent Variable	Direct Effects (T)	Indirect Effects (T)	Total Effects	VAF	Result
GNM	TRUST	EFF	0.028 (0.710)	0.135 (4.580)	0.163	0.828	Full mediation
MM			0.018 (0.523)	0.073 (2.705)	0.091	0.802	Full mediation
KOL			0.066 (1.777)	0.072 (2.459)	0.138	0.522	Partial mediation
GNM	TRUST	EFF	0.04 (0.959)	0.131 (4.603)	0.171	0.766	Partial mediation
MM			−0.049 (1.390)	0.070 (2.751)	0.022	3.182	Full mediation
KOL			−0.036 (0.922)	0.070 (2.477)	0.033	2.121	Full mediation

## Data Availability

All scales used for the study are available from the corresponding author upon reasonable request.

## Appendix A

**Table A1:** Scale of the Study.

Constructs		Measurement Items
Government New Media	GNM1	Official accounts of government and publicity departments.
	GNM2	Accounts of health committees and CDC at all levels.
Mass Media	MM	Accounts of mass media such as radio, newspaper, television.
Key Opinion Leaders	KOL	Famous experts in the health care field (e.g., Nanshan Zhong, Wenhong Zhang, Lanjuan Li).
Professional Organizations	PO1	Famous universities, laboratories, research centers.
	PO2	Hospitals, community health centers.
	PO3	Science and technology media company social media account (e.g., Dr. Dingxiang).
	PO4	International Organizations (e.g., WHO) social media account.
Personal Networks	PN	Relatives, close friends, neighbors, etc.
Trust in Government Approval of Vaccines	TRUST1	The information I receive about vaccines from the government is reliable and trustworthy.
	TRUST 2	Government will communicate honestly about the possible health effects of a COVID-19 vaccine.
	TRUST 3	If a COVID-19 vaccine is found to be a health risk, government will openly and honestly inform the public.
	TRUST 4	I trust government to make safety a priority when approving COVID-19 vaccines.
	TRUST 5	Government has a history of only approving vaccines once they have been proven safe and effective.
	TRUST 6	I have confidence that government approved COVID-19 vaccines will be safe and effective.
	TRUST 7	I have confidence in the government to properly assess the safety and effectiveness of COVID-19 vaccines.
Perceived Effectiveness of Vaccines	EFF1	If my child receives a COVID-19 vaccine, he/she will not get sick from COVID-19.
	EFF2	Vaccination is good because it will make me less worried about my child becoming sick from COVID-19
	EFF3	If my child receives a COVID-19 vaccine, he/she will not get very sick from COVID-19 (or even be infected by the virus)
	EFF4	The complications of COVID-19 will decrease if my child is vaccinated and then infected with the coronavirus.
Side Effects (safety) of Vaccines	SAV1	I think the COVID-19 vaccine has undergone enough testing to be safe.
	SAV2	I am not worried at all about adverse side effects of a COVID-19 vaccine.
	SAV3	I am not concerned at all about potential adverse effects from accepting a COVID-19 vaccine.
	SAV4	I am not concerned at all about potential negative long-term side effects from a COVID-19 vaccine.
Vaccination Intentions	VI1	If a COVID-19 vaccine became available I would accept the vaccine for my child/children.
	VI 2	I will certainly receive the COVID-19 vaccine when and if it is approved by government.
